# Leukemic appendix with clinical presentation that mimics acute appendicitis

**DOI:** 10.1007/s12308-026-00681-x

**Published:** 2026-02-05

**Authors:** Oren Weiss, Yanhua Wang

**Affiliations:** Department of Pathology, Albert Einstein College of Medicine/Montefiore Medical Center, Bronx, NY USA

**Keywords:** Acute myeloid leukemia, Myeloid sarcoma, Extramedullary leukemia, Leukemic appendix, *NPM1* mutation, Acute appendicitis mimic

## Abstract

Acute myeloid leukemia (AML) is a malignant neoplasm characterized by the uncontrolled, clonal growth of hematopoietic cells. It is the most common acute leukemia in adults, with a median age at diagnosis of 69 years and an incidence of 4.2 cases and mortality of 2.7 per 100,00 people per year in the USA, as reported by SEER (2025) and Shallis et al. (*Blood Rev* 36:70–87, 2019). With the advent of more detailed molecular studies, next-generation sequencing, and novel therapies, the prognosis for patients with AML has significantly improved, as discussed by Shimony et al. (*Am J Hematol *98(3):502–526, 2023). However, there are numerous complications of treatment and progression of disease that can affect patients’ quality of life and overall survival, as detailed by Khwaja et al. (*Nat Rev Dis Primer* 2(1):1–22, 2016). Below, we describe an unusual complication of AML in a woman who previously achieved complete remission.

## Case description

A 52-year-old woman with a past medical history of asthma, obesity (BMI > 35), abnormal uterine bleeding, anemia, and acute myeloid leukemia (AML, status post chemotherapy) presented to the emergency department for sudden onset right lower quadrant (RLQ) pain. On admission, she was found to have fever, tachycardia, and tenderness to palpation in the right lower quadrant without rebound or guarding. Laboratory testing was notable for leukocytosis (87.3 × 10^9^ cells/L) with an elevated neutrophil count (9.5 × 10^9^ cells/L) and left shift (immature granulocyte count 4.73 × 10^9^ cells/L), anemia (Hb 7.9 g/dL, Hct 21.2%), thrombocytopenia (29 × 10^9^ platelets/L), and increased circulating blasts (24% on manual differential).

She was diagnosed 1 year prior to presentation with AML with monocytic differentiation via flow cytometry of peripheral blood showing a population of 18.4–24.3% abnormal cells positive for CD13, CD33, CD34, CD117, and CD123; bone marrow aspirate demonstrating a lack of erythroid and megakaryocyte lineages and a large population of atypical monocytes; and normal cytogenetics and FISH. Further genetic testing found the blasts were positive for *NPM1*, *DNMTA3*, and *IDH1* gene mutations and negative for *FLT3*-ITD. She was treated with induction therapy 7+3 chemotherapy regimen (cytarabine and idarubicin) with four cycles of consolidation with high-dose cytarabine chemotherapy leading to complete remission on post-treatment bone marrow biopsy. One month prior to the current presentation, she was admitted for interventional radiology–assisted bone marrow biopsy due to persistent pancytopenia. Flow cytometry from bone marrow aspirate showed 41.5% blasts positive for CD33, CD45, and CD117 suggesting relapsed AML. Due to the presence of *IDH1* mutation, the patient elected to enroll in a clinical trial for an experimental oral mutant *IDH1* inhibitor.

On evaluation in the emergency department, CT scan demonstrated appendiceal enlargement (1 cm at the largest extent) with fat stranding suggestive of acute appendicitis.

Repeated bone marrow evaluation found a hypercellular marrow (> 90%) with reduced trilineage hematopoiesis; 32.7% abnormal blast cells positive for CD11c, CD33, CD45, and CD117; and 35.3% monocytes. The patient was treated for suspected differentiation syndrome, a known complication of *IDH1* inhibitor drugs suggested by the monocytic predominance in the bone marrow biopsy, and a laparoscopic appendectomy was performed 4 days following the initial presentation [1].

**Fig. 1:**
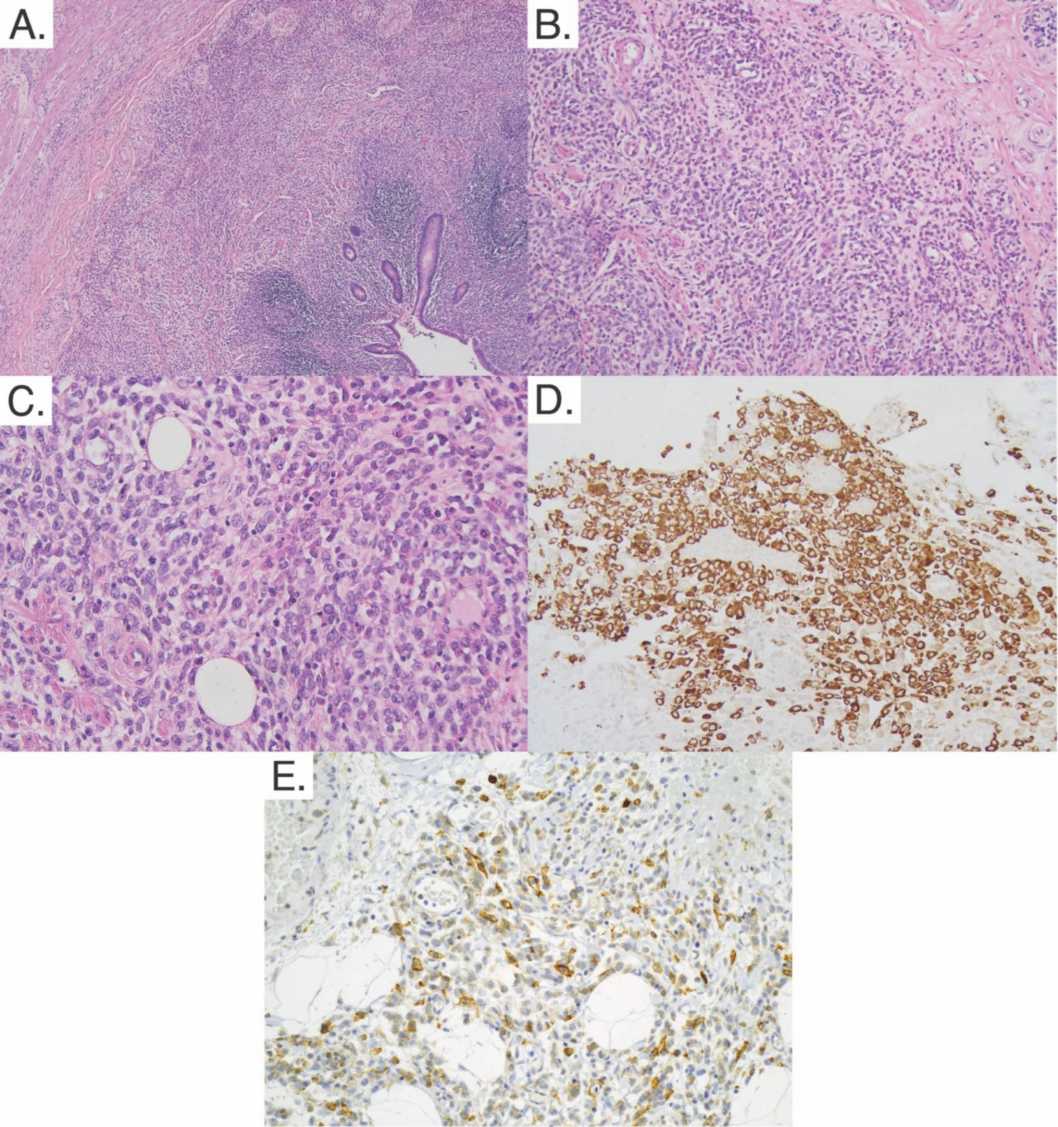
Cross section of the appendix. **A** H&E low power (×40) showing wall infiltration distinct from appendiceal lymphoid tissue. **B** H&E intermediate power (×100) showing atypical cells, mitotic figures, and variable nuclear to cytoplasmic ratio. Note the lack of neutrophilic infiltration. **C** H&E high power (×400) showing further detail. **D** MPO stain intermediate power (×200) showing myeloid-lineage leukemic cells in the tissue. **E** CD117 stain intermediate power (×200) showing immature myeloid-lineage leukemic cells in the tissue

Intraoperative assessment characterized the appendix as acutely inflamed, distended, non-purulent, non-perforated, and not associated with any peri-appendiceal inflammation or free fluid in the pelvis. On pathological exam, the appendix measured 7.0 × 1.5 × 1.4 cm with the appendiceal wall up to 0.4 cm in thickness. The serosa and mucosa were tan-red, and the lumen of the appendix was filled with hemorrhagic fecal material. On microscopic exam, there was extensive infiltration of the mucosa, submucosa, and muscularis layer with atypical medium to large cells with irregular nuclear contours, delicate chromatin, and high nuclear to cytoplasm ratio. Additionally, numerous mitotic figures were noted. Notably, inflammatory changes associated with typical appendicitis were not seen (e.g., neutrophilic predominance). The atypical cells were characterized as myeloid precursors. Immunohistochemical stains confirmed this, as they displayed a similar staining phenotype to the leukemic cells in the bone marrow: they were positive for CD33, CD117, and myeloperoxidase (MPO), while being negative for CD3, CD20, and CD34 (Fig. 1). This analysis led to the diagnosis of appendicitis caused by acute myeloid leukemic cell infiltration.

Following appendectomy, the patient was discharged from the hospital with a plan for further chemotherapy. Flow cytometry of peripheral blood found circulating blasts (19%) and a rising *FLT3*-ITD mutation. Ten months after the initial presentation for abdominal pain, despite several rounds of chemotherapy, a repeat bone marrow biopsy found a high percentage of myeloblasts (> 60%). The patient’s disease was resistant to salvage chemotherapy, and she passed away 2 months later.

## Discussion

Extramedullary infiltration of leukemic cells into other tissues is a rare complication of AML, which can cause varied clinical syndromes depending on the involved organ or tissue [2, 3]. Appendiceal involvement has been documented in fewer than 20 reported cases, including our case (Table 1). In autopsy studies before the advent of modern chemotherapy, leukemic infiltration into gastrointestinal tissue was found in 14.8 to 25% of cases, possibly representing a late consequence of untreated disease [4]. One manifestation of this leukemic invasion is referred to as myeloid sarcoma, defined as a tumor composed of leukemic cells that disrupts normal tissue architecture [5]. This definition has been noted to be somewhat ambiguous; although the WHO definition explicitly distinguishes between myeloid sarcoma and generalized leukemic cell infiltration into tissues (which does not necessarily form a solid tumor or affect the structure of normal tissue), these terms are often used synonymously [6]. Myeloid sarcomas are uncommon, occurring in only about 2 to 9% of patients with myeloid leukemia and related myelodysplastic syndromes, and can occur with or without bone marrow involvement [7].

**Table 1:** Previously reported cases of leukemic cell infiltration into the appendix

Patient characteristics (age, gender)	Diagnosis	Initial presentation or relapse	Genetic or immunohistochemical characterizations	Treatment	Overall course	Reference
33, male	AML	Developed appendicitis during initial induction chemotherapy	N/A	Vincristine, Adriamycin, l-asparaginase, prednisone	N/A	[8]
77, female	Acute promyelocytic leukemia (AML M3)	Initial presentation	MB2+, chloroacetate esterase+, CD43+, CD3-, CD20-, CD45RO-, CDw75-	Retinoic acid (refused by patient)	Died 1 month after admission	[9]
71, male	AML M2	Initial presentation	**Cells infiltrating the appendix**: CD45rb+, CD34+, CD4-, CD38-, CD56-, CD117- **Bone marrow aspiration:** CD4+, CD11b+, CD13+, CD33+, CD34+, CD38+, CD56+, CD117+, HLA DR+ **Genetics**: 46 XY del(12)[6/8] and 46 XY [2/8]	Enocitabine, daunorubicin hydrochloride, 6-mercaptopurine riboside, idarubicin hydrochloride, cytarabine	Died 49 days after admission	[10]
29, male	AML	Relapse	N/A	N/A	N/A	[11]
9, female	AML M4	Initial presentation	**Cells infiltrating the appendix**: Chloroacetate esterase+, CD43+, CD68+, CD117+, lysozyme+, MPO+, CD3-, CD20- **Bone marrow aspiration**: CD2 (dim), CD13+, MPO+, HLA DR+, CD3-, CD7-, CD10-, CD19-, CD79a-	Chemotherapy	Remission up to 27 months after admission	[12]
29, male	AML M2	Relapse	**Cells infiltrating the appendix**: CD43+, CD68+, lysozyme+, MPO+, CD3-, CD20- **Bone marrow aspiration:** CD7+, CD11c+, CD13+, CD33+, CD34 (dim), MPO+, HLA DR+	Chemotherapy, radiotherapy	Alive with persistent AML 16 months after initial diagnosis	[12]
75, male	AML M2	Relapse	MPO+, CD43+, CD34+	Cytarabine	Died 19 days after admission	[13]
31, male	Acute promyelocytic leukemia	Developed appendicitis during initial induction chemotherapy	**Cells infiltrating the appendix**: MPO+, CD34- **Bone marrow aspiration**: “expression of mostly myeloid markers”, CD34- **Genetics**: t(15;17), partial deletion 3p, 3q, 1p, paracentric inversion of 1p	Idarubicin, cytarabine, all trans-retinoic acid (induction) Arsenic trioxide	Remission	[14]
68, female	AML del 7(q22q32)	Relapse	CD117+, MPO+	Cytarabine	N/A	[15]
59, female	AML with mutated NPM-1	Initial presentation	*NPM1* mutation, *FLT3* ITD wild-type	Idarubicin, cytarabine	Died 185 days after admission	[16]
43, female	Acute promyelocytic leukemia (AML M3)	Initial presentation	MPO+, CD68+ **Genetics**: t(15;17)q(22;12), PML-RARA fusion	Daunorubicin, cytarabine, all trans-retinoic acid	Remission up to 48 days after admission	[17]
10, female	AML M2	Initial presentation	N/A	Decitabine, cytarabine	Remission up to 2.5 years following completion of therapy	[18]
28, male	AML	Initial presentation	N/A	Cytarabine	Died 1 month after admission	[19]
9, female	AML M5A	Initial presentation	CD45+, BCL2, MPO+	N/A	N/A	[20]
7, male	AML	Initial presentation	CD33+, CD117+, CD163+, MPO+, CD4-, CD34-	Cytarabine	N/A	[21]
57, male	AML transformed from RAEB-2	Relapse	MPO+, CD68+, CD16+, scattered CD117+ and CD34- negative blasts	N/A	N/A	[22]
31, female	Acute promyelocytic leukemia	Initial presentation	MPO+	N/A	Died postoperatively (appendectomy)	[23]
52, female	AML with mutated *NPM1*	Relapse	CD33+, CD117+, MPO+, CD3-, CD20-, CD34- **Genetics**: *NPM1* mutation, *DNMT3A* mutation, *IDH1* mutation, rising *FLT3-*ITD mutation clone following appendectomy	Decitabine/midostaurin, venetoclax/azacitidine, fludarabine/cytarabine/G-CSF/Idarubicin, gilteritinib	Died 389 days after admission	This case

The pathophysiology underlying the invasion of leukemic cells into tissues is not well understood. There are numerous pathogenic mutations that are associated with myeloid sarcoma and extramedullary leukemic cell infiltration, including *NPM1*, *DNMT3*, and *IDH1*, among others [24–26]. Symptoms of myeloid sarcoma arise from disruption of the normal functioning of the tissue they invade; the most common sites of extramedullary infiltration include soft tissue, skin, and lymph nodes [25]. Appendicitis caused by extramedullary invasion of neoplastic myeloid cells has been reported in the past and can occur as the initial presenting condition or in the setting of a relapse (see Table 1) [8–23]. The prognostic relevance of extramedullary infiltration by leukemic cells has been disputed: some studies have pointed to inferior outcomes for patients with leukemic infiltration while others have suggested there is no effect of the presence of extramedullary manifestations of AML on overall survival when compared to patients with medullary involvement alone [3, 27–29]. Treatment of extramedullary AML in the context of relapse, as in this case, involves systemic chemotherapy, with the addition of localized radiotherapy in the case of myeloid sarcoma, or surgery in some cases requiring emergent decompression [3, 6, 30, 31]. Some recent advances on targeted therapies for genetic mutations could point towards better management of acute myeloid leukemia and aid in the treatment or prevention of leukemic infiltration into other tissues [32, 33].

To conclude, we present an unusual case of appendicitis in a 52-year-old woman caused by invasion of the appendiceal tissue by neoplastic myeloid cells in the setting of relapsed AML. This report adds to the body of literature regarding the potential of leukemic cells to invade tissue and the clinically relevant consequences of the disruption of normal organ function.

## Data Availability

No datasets were generated or analyzed during the current study.
